# Perceived effectiveness: simulation teeth in dental education

**DOI:** 10.1186/s12909-026-09672-z

**Published:** 2026-06-29

**Authors:** Alice M. G. Cheadle, Ronan Bagry, William M Palin, Phillip L Tomson, Renate LEP Reniers, Mohammed A Hadis

**Affiliations:** 1 Department of Dentistry, College of Medicine and Health, University of Birmingham, Birmingham, UK; 2 Birmingham Medical School, College of Medicine and Health, University of Birmingham, Birmingham, UK; 3 Institute for Mental Health, University of Birmingham, Birmingham, UK

**Keywords:** Simulation, Dental education, Typodonts, 3D-printing

## Abstract

**Background:**

Typodont simulation is a key part of undergraduate dental training globally, allowing prospective dentists to repeatedly practice the necessary skills to safely and effectively care for their future patients. Anecdotally, and in previous small studies, typodonts fall short when compared to what they aim to replicate: human teeth. This study aimed to evaluate opinions on the effectiveness, use, advantages and disadvantages of typodonts.

**Methods:**

This study gathered quantitative and qualitative data from 338 dentists and dental students using an online questionnaire, evaluating their opinion on the effectiveness of typodont simulation. Likert scales to 32 questions collected opinions on effectiveness of various typodont attributes such as tactile feedback and appearance. Responses were analysed to examine differences in opinion across participant groups using the appropriate parametric and non-parametric tests; free-text responses were analysed thematically.

**Results:**

Typodonts were used extensively in all surveyed institutions. They were considered to be moderately effective and readily available for repeated practice of direct and indirect preparations. Improvement is required in several key areas: caries simulation, anatomical layering and endodontic simulation, particularly in relation to material properties related to tactile realism. There were varying opinions on cost and environmental impact when comparing methods of manufacture. Standardisation and customisation of typodonts for education was valued by all.

**Conclusion:**

Anatomical layering, tactile realism and caries are important factors to students and dentists in simulation education. Traditionally manufactured typodont were used by the majority of those surveyed but lack key qualities such as realistic tactile feedback required for effective simulation of human teeth, simulation of caries and root canal anatomy. There is potential for more modern methods of manufacture such as 3D-printing to assist in this need for better representation of the complex anatomy and feel of teeth, positively impacting both patient safety and dental education, yet these still fall short in realistic haptic feel and caries simulations. Alongside these features, environmental, financial and availability should be considered.

## Introduction

The use of human teeth for dental simulation in education has declined owing to well reported issues such as lack of standardisation, governance issues with respect to managing human tissues, limited supply and increased cross-infection risk [1, 2] alongside the development of cheap mass manufactured typodonts. Now, dental institutions rely heavily on the use of artificial models of human teeth, known as ‘typodonts’, to train undergraduates in the necessary technical skills for clinical dentistry [3–5]. Simulation-based-education is commonplace in dentistry to allow safe, risk-free and repeated practice of clinical skills requiring dexterous skills prior to treating patients [6]. This simulated experience of procedures such as cavity and crown preparation, caries excavation and endodontic treatment forms a key part of undergraduate and postgraduate dental training with millions of typodonts used per year worldwide. Despite previously identified specific limitations in physical typodonts, they make a significant contribution to dental education across the world [3, 5].

**Fig. 1:**
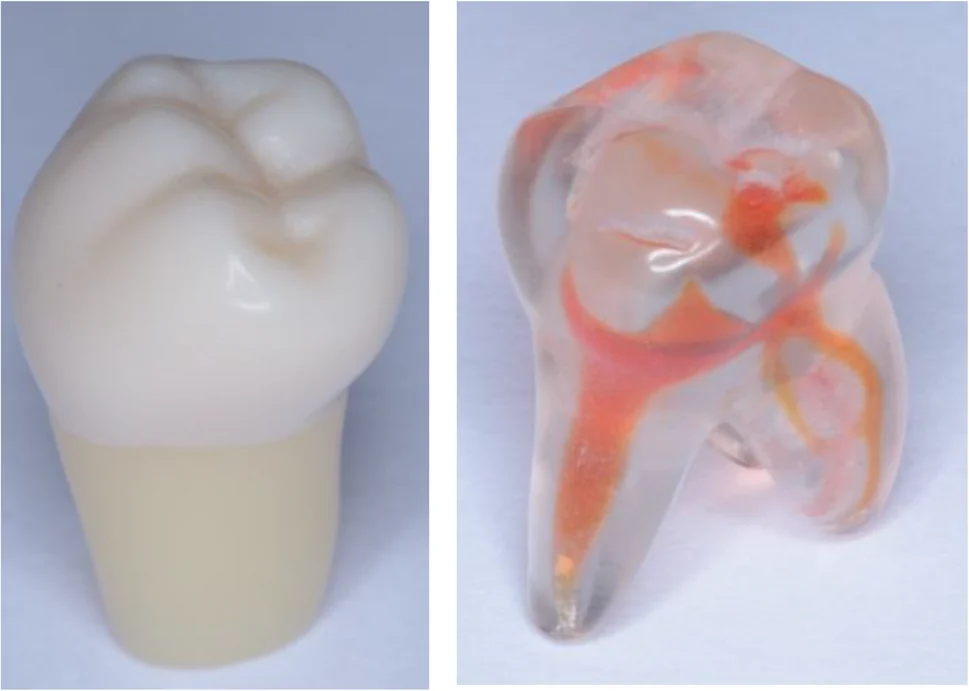
Photographs of two commercially available typodonts. Left: two-layered typodont representing enamel (white) and dentine (cream) (Frasaco GmbH, Germany). Right: Transparent 3D printed upper molar tooth designed for endodontic teaching with the root canal space produced in red (TrueTooth, PlanB Dental,, USA)

Typodonts vary by design complexity and educational focus, as well as manufacturing method ergo cost. Typodonts vary in basic structure: some may be ‘monoblock’ and formed of a single material, whilst others may have layers of distinct colours and or materials to recreate the anatomical layers of the human tooth - enamel, dentine and pulp (Fig. 1). Commercial manufacturers most commonly mass produce plastic typodonts utilising injection moulding. More recently, with the development of 3D-printing, some newer more sophisticated typodonts are produced using vat polymerisation (e.g. stereolithography, digital-light-processing) enabling the use of computer-aided design [3, 7]. Some well-equipped institutions have published work on fabricating in-house 3D-printed typodonts for pre-clinical simulation, although this is not yet commonplace [8–10]. No matter the production method, typodont are single-used items which contribute to non-biodegradable waste [11].

Whilst many manufacturers keep materials and manufacturing methods undisclosed or patented, some research groups openly publish prototypes of more advanced typodonts with improved educational outcomes [12], particularly in the field of Endodontics where 3D-printing has been used to attempt to print complex root anatomy [13–15]. Despite the wide variety of commercially available typodonts, several studies have reported individual model limitations including expense, poor tactile feedback [16], lack of customisation [17, 18] and poor cleanliness [19].

### Aims

It is essential that typodonts support the development of clinical skills and foster student engagement in their learning, offering a realistic and immersive training experience. This study aimed to evaluate opinions of dental students, qualified dentists and educators in multiple dental institutions on multiple themes: appearance, tactile feedback, educational experience, caries texture, root canal system realism, direct and indirect preparation simulation and ethical / environmental implications of traditional typodonts. The secondary aim is to identify how typodont development could enhance education within dental institutions.

### Hypothesis

It is hypothesised that dental students and qualified dentists will report limited perceived effectiveness of traditional typodont simulation in their appearance, tactile feedback, educational experience, simulation of caries, root canal treatment and direct and indirect preparation. It is further hypothesised that these opinions will be similar across multiple institutions, and that student and qualified dentist responses will be aligned.

## Methods

### Ethics

Ethics approval was granted by the University of Birmingham Science, Technology, Engineering and Mathematics Committee (ERN_3608-Jan2025). All participants were asked to consent prior to participation following an introduction to the aims of the questionnaire. Responses were anonymised; student participation was optional and not a requirement for academic progress and no incentives were provided for participation in the study.

### Participants

Participants included pre-clinical and clinical dental students from any year of study, qualified dentists and clinical dental educators. Participants all had experience of using typodonts and extracted human teeth for simulation. Participants were recruited within the University of Birmingham (UoB) via email to all staff and students. All clinical UoB staff were asked to email the questionnaire to their professional networks colleagues in dental institutions who they knew to be involved in undergraduate dental education in order to recruit staff and students from other institutions. A hyperlink and QR code were used to link participants to an online questionnaire. A period of two weeks of repeated reminders asking for participation was carried out.

### Questionnaire design

Both quantitative and qualitative data were captured using a questionnaire in an online MS-Form (Microsoft, USA). The questionnaire (Table 1) comprised 34 questions (in English), this was developed and discussed amongst the authors and piloted amongst 5 Clinical Lecturers and 5 students at the University of Birmingham, UK. Feedback was gathered on clarity, relevance, length, structure and flow with appropriate adjustments made. An introduction to the questionnaire defined the classifications of typodont teeth to reduce misinterpretation by the participant which included explanation of: traditional and 3D-printed manufacture, single-layer or multi-layer anatomy and commercial and in-house manufacture. The first 32 questions in the questionnaire proposed a stem with a Likert-scale [1–5], allowing for statistical analysis and enabling a graded opinion on importance, realism, effectiveness and concern. A middle response of ‘3’ indicated neutrality, whilst a score above or below ‘3’ indicated positivity or negativity, respectively. Free-text responses were utilised in questions 33 and 34. External participants completed three extra questions on typodont varieties in use in their institutions (Table 2).

**Table 1:** Internal questionnaire to evaluate the effectiveness, use, advantages and disadvantages of typodonts. External participants completed predominantly the same questionnaire with the following changes: ‘Birmingham’ changed to ‘your institution’ in questions 3-8 and question 31 changed to ‘Does the volume of typodont teeth used annually at our institution bother you from an environmental point of view? Some additional external questions asked are given in Table 2

How realistic do you find the typodont teeth you have predominantly used in terms of the following?1 = very unrealistic, 5= very realistic	1	Appearance compared to human teeth (aesthetic and anatomical)
	2	Tactile feedback during drilling
How effective are the typodont teeth used in Birmingham at preparing individuals for the following operative procedures in a clinical environment?1 = not very effective at all,5 = very effective	3	Direct restoration cavity preparation (e.g. composite, amalgam)
	4	Caries detection
	5	Caries removal
	6	Indirect restoration preparations (e.g. crowns, onlays, conventional bridges)
	7	Simulating adhesive dentistry (e.g. adhesive onlays, resin retained bridges)
	8	Root canal treatment
How important to you are these common advantages of typodont teeth compared to human teeth?1 = not important at all,5 = very important	9	No ethical concerns with use
	10	Readily available for repeated practice
	11	Consistent material quality
	12	No cross-infection risk
	13	Standardisation of educational experience
	14	Standardisation of exercises used for examinations/competencies
How important to you are these common disadvantages of typodont teeth compared to human teeth?1 = not important at all,5 = very important	15	Unrealistic tactile feedback, not similar to human tooth
	16	Limited anatomical accuracy
	17	Environmental impact of plastic models
	18	Lack of variation between samples
	19	High cost of advanced models.
	20	Adhesive dentistry (e.g. adhesive onlays, resin retained bridges)
	21	Not replicating real patient cases
What factors are most important to you when using a typodont tooth for simulation of operative work?1 = not important at all,5 = very important	22	Presence of pulp
	23	Root canals for endodontic training
	24	Anatomical layers (enamel and dentine)
	25	Realistic tactile feedback to drilling
	26	Natural tooth colour
	27	Simulation of caries
	28	Case-specific typodonts (e.g. missing restorations, tooth wear).
	29	Standardisation for consistent assessment
	30	Environmentally friendly, waste limiting
31 In your opinion, does the use of over 10,000 plastic teeth annually in this institution bother you from an environmental point of view? 1 = not concerned at all, 5 = very concerned		
32 3D printing resins can be made from petroleum derived chemicals and (in their liquid form) can be harmful to aquatic life and/or can cause human allergic skin reactions and eye damage. Does this concern you? 1 = not concerned at all, 5 = very concerned		
33 What suggestions would you make for designing the perfect typodont tooth? (free text answer)		
34 Do you have any experience of any more advanced typodont teeth that you can describe and give an opinion on? (free text answer)		

**Table 2:** Additional questions given to external participants to ascertain usage of varied typodonts

Do you know what type of typodont teeth you use/have predominantly used in simulation teaching?	Commercially available traditional typodont teeth
	Commercially available 3D-printed typodont teeth
	Both traditional and 3D printed
	In-house-made typodont teeth
	I don't know
Which types of typodont teeth have you used predominantly?	Single layer (monoblock)
	Multilayer
	I don't know
Does your university (or you) print typodont teeth for educational use?	Yes
	No
	I don’t know

### Statistical analysis

Data was collected over a two-week period in March 2025 and was exported to Microsoft Excel (Version 2503, 2025; Microsoft, USA). Likert response data from the 32 questions were individually assessed using histograms and skewness calculations. Analysis was intended to highlight descriptive trends rather than conclusive statistical associations in this exploratory study that included distinctive themes. Appropriate statistical tests were applied using statistical software (SigmaPlot Version 15.0, Systat Software Inc, USA). Post-hoc Tukey comparisons were applied to normally distributed data and Mann Whitney U tests were applied to non-normally distributed data. Differences (*p* < 0.05) across experience groups were tested using the Kruskal-Wallis test with Dunn’s post-hoc pairwise comparisons. Free-text responses were analysed thematically using Bruan and Clarke’s approach [20].

Power calculations for the required number of responses to enable statistical significance (*p* < 0.05) were calculated using G* Power calculations resulting in a minimum of 98 participants (G* Power analysis) (Version 3.1.9.7, HHU Dusseldorf, Germany) [21]. However, a larger sample was aimed for to allow subgroup analyses.

## Results

This study gathered quantitative and qualitative data from 338 dentists and dental students across 30 institutions, evaluating their opinion on the effectiveness of typodont simulation. Analysis of both quantitative and qualitative data allowed an in-depth exploration into the effectiveness of typodont simulation.

### Questionnaire response

An initial 222 internal (UoB) and 131 external responses were screened for suitability for entry into the study (Table 3). 14 responses were removed due to: lack of consent, no involvement in clinical dental education or no previous use of typodonts. A final total of 339 responses were analysed. A total of 302 free-text comments were collected, comprising 208 and 94 in response to Questions 33 and 34 respectively (Table 1). External responses were received from participants in UK (44), Europe (16) (Germany, Portugal, Spain, Netherlands, Italy, France and Belgium), New Zealand (26), Hong Kong (4), India (4), (UAE (6), Jordan (1), North America (2), Egypt (1). 16 participants did not declare their country.

**Table 3:** Number of responses received internally (UoB) and externally, further sub-categorised by experience

	Internal Responses *n* (%)	External Responses *n* (%)
Responses removed – 14 (4%)	3	11
No consent (0.56%)	1	1
Not clinically trained/in training (1.42%)	2	3
No previous use of typodonts (2%)	-	7
Total responses (339)	**219**	**120**
Dental undergraduate (student) (58%)	156 (71%)	41 (34%)
Qualified Dentist (QD) (16%)	30 (14%)	23 (19%)
Qualified Dentist and Educator (QDE) (26%)	33 (15%)	56 (47%)

### Typodont use

UoB typodont purchases for the 2023/34 academic year for undergraduate and postgraduate training totaled £18,091.70. The vast majority (approximately 11,000 in 2023/24) of typodonts used in undergraduate prosthodontic training are traditionally manufactured monoblock using injection moulding (KaVo Dental GmbH, Germany). Additional commercially available 3D-printed typodonts are purchased in smaller quantities (200 in 2023/24) for use in undergraduate endodontic specialty teaching (TrueTooth, USA), alongside extracted human teeth with ethical approval.

**Fig. 2:**
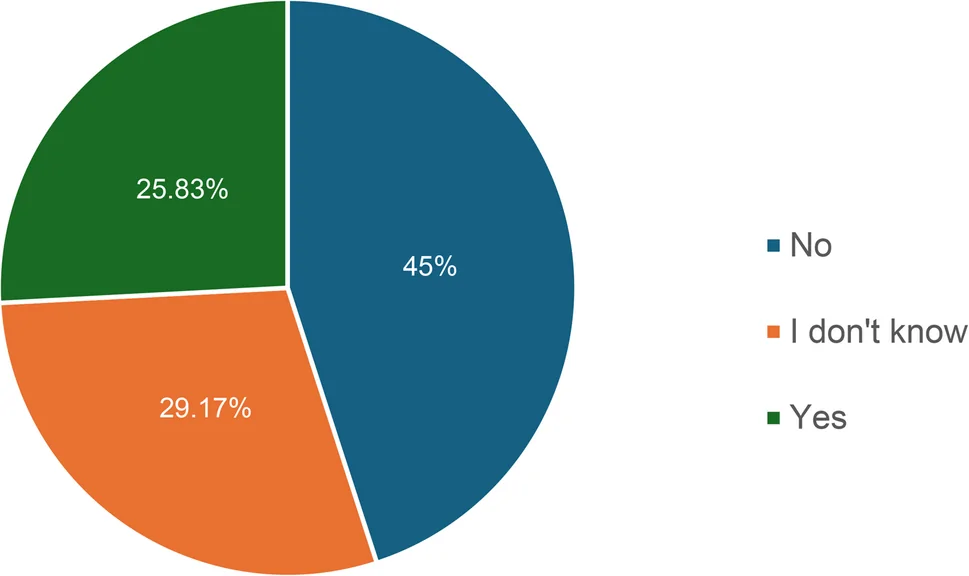
Pie chart depicting the response to the external question: ‘Does your university (or you) print typodont teeth for educational use?

Within the external institutions, 85% of the typodonts used in simulation teaching were commercially available and traditionally manufactured; commercially available 3D-printed typodonts were used by 5%, and a single response (< 1%) was given for the categories ‘both traditional and 3D-printed’ and ‘in-house made typodonts’. Some did not know what was in use at their institution highlighting limited awareness (8%), 70% of these responses were dental students. Monoblock typodonts were used by 90%, 5% used multi-layer and 5% participants did not know. The proportion declaring predominant use of in-house 3D-printed typodonts’ (0.83%) was a fraction of the 25.83% of responses declaring 3D-printing of typodonts in their institution (Fig. 2), suggesting that – as in Birmingham – 3D printing is an adjunct to traditional resources perhaps with specialised requirements such as endodontics and cariology.

### Appearance

**Fig. 3:**
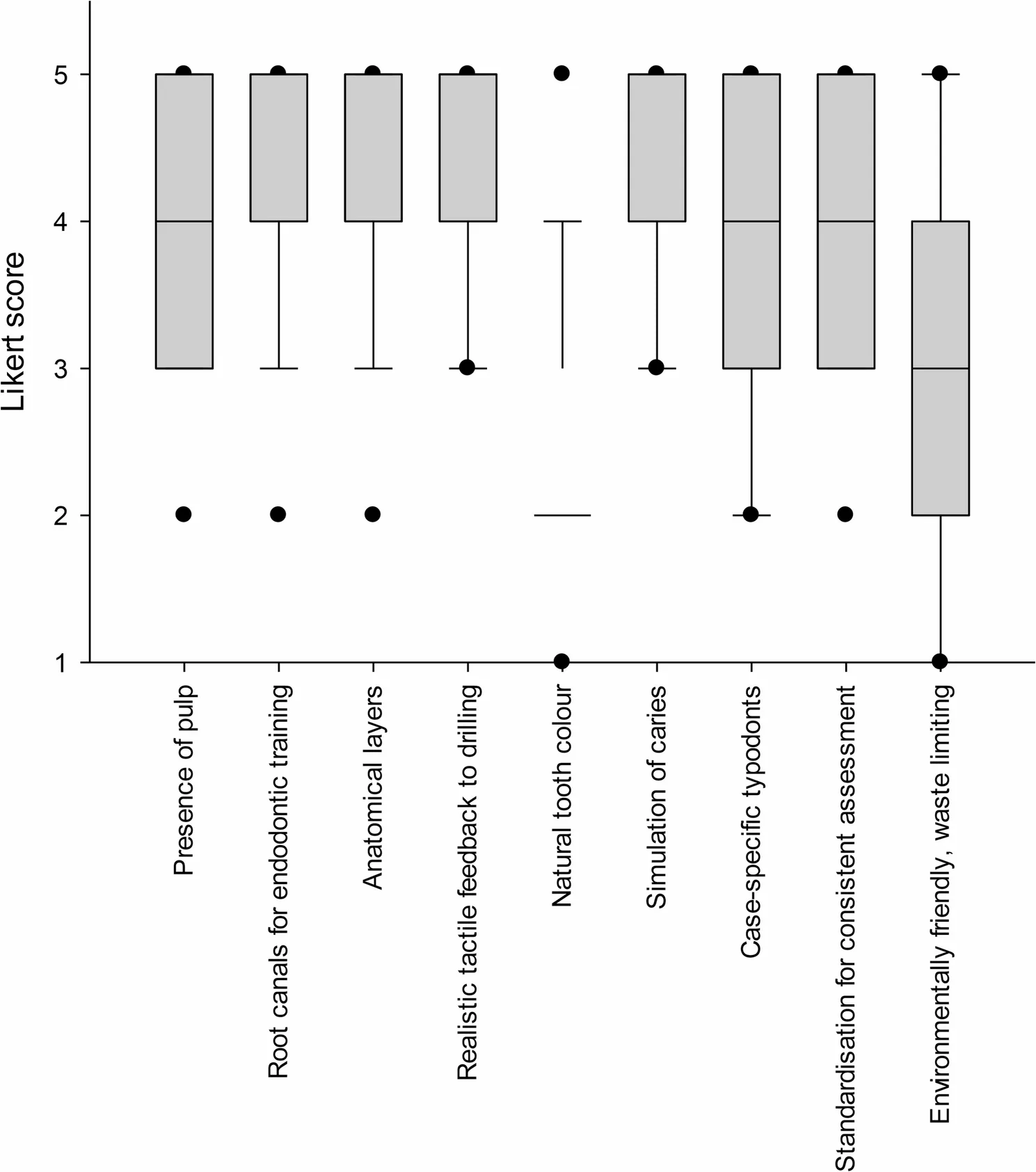
Box and Whisker plot depicting the overall average responses to Questions 22–30: ‘What factors are most important to you when using a typodont tooth for simulation of operative work?’. Participants ranked importance using a 1–5 Likert scale: 1 = not important at all, 5 = very important. The box depicts the middle 50% of data, whiskers extending from the box the minimum and maximum scores excluding outliers (dots)

There was consistent agreement on the importance of tooth layer simulation in the free-text responses across all groups when asked *‘What suggestions would you make for designing the perfect typodont tooth?’*, for example: *‘Differential layers simulating the enamel*,* dentine and pulp with the associated tactile feedback’*. Whilst the overall rating for the broad anatomical and aesthetic realism of typodonts was neutral (M = 3.07 ± 0.92), further questions into more detailed features revealed that the presence of anatomical layers simulating those of enamel and dentine was important (M = 4.08 ± 0.93) (Fig. 3).

Despite natural tooth colour being scored with lower importance overall (M = 2.43 ± 1.21), colour was mentioned as useful in the context of being able to differentiate between the layers of teeth. One comment suggested an accessory use of colour to assist in indirect preparation simulation: *‘Crown prep typodont where colour of plastic changes to indicate sufficient reduction’*. The disadvantage of typodonts not replicating real patient cases (M = 4.02 ± 0.98), and the importance of case-specific typodonts (M = 3.72 ± 1.09) were valued highly by all participants.

### Tactile feedback

The call for distinct typodont layering was clear; some free-text responses elaborated asking for layers to *‘feel’* different in their *‘hardness’*. However, there were many comments to suggest this tactile feedback was not satisfactory: ‘*They [typodonts] are often made of plastic*,* this limits certain tactility provided by ‘real’ teeth’*, *‘I struggle to know how to interpret the active feedback I’m getting’*, *‘Typodont teeth feel completely different to real teeth when drilling’* and *‘my first filling felt nothing like the teeth I had practised on’.*

Lack of realism from current typodont offerings was echoed in quantitative data: all groups ranked the realism of tactile feedback poorly (M = 2.25 ± 0.89). All participants also agreed on the importance of realistic tactile feedback with a mean score of 4.39 (± 0.83) (Fig. 3); a Mann-Whitney U test indicated no statistically significant difference between the students (Mdn = 5) and qualified dentists (Mdn = 5) (*U* = 23063.50, *p* = 0.413).

### Caries

Caries was the most frequently mentioned in the free text comment, and in 26% (54 comments) of the responses to Question 33. One comment linked caries preparation to teaching as a key and difficult clinical concept: ‘*The caries should be of a variety of depths/shapes/sizes in different teeth so that you can practice more complex restorations and as a student be able to more easily decide between direct and indirect restoration based on cavity size/shape’*. A succinct message for a perfect typodont tooth was: ‘*Flexible design*,* caries driven’.*

Given that the predominant typodont in use is monoblock (90% externally and approximately 98% internally), caries detection was unsurprisingly rated as ineffective (M = 1.52 ± 0.83) as well as caries simulation (M = 1.66 ± 0.92). Internal scores were analysed using a Kruskal-Wallis H test to examine opinion on effectiveness of caries simulation across groups of increasing level of experience: BDS2, BDS3, BDS4, BDS5, QDs and QDEs; there was a statistically significant difference in opinion between groups (*H* (5) = 17.46, *p* = 0.004), with a significant decline in satisfaction as experience increased. Post-hoc pairwise comparisons using Dunn’s method indicated that BDS2 differed significantly from BDS4 (*p* = 0.004) and BDS5 (*p* = 0.037), whilst more experienced groups did not differ significantly from each other (*p* > 0.05). BDS2 was the only group to have not experienced clinical caries removal on patients.

Despite poor effectiveness scores, caries simulation was consistently scored with foremost importance, with an overall mean score of 4.18 (± 0.90) and a Mann-Whitney U test result indicating there was not a significant difference between internal (Mdn = 4) and external (Mdn = 4) participants (*U* = 12,793.50, *p* = 0.68).

### Endodontics

More advanced typodonts used for endodontic training were mentioned to be beneficial by many students: *‘Used printed teeth with pulp material on an endo course. Pretty good*,* cutting feels different to natural teeth but anatomically better’*. This was not limited to undergraduates with qualified dentist comments including: *‘Use of transparent teeth with pulp chamber for root canal teaching*,* feel these are a good way to learn and visualize pupal chamber’*, ‘*We used 3D printed teeth for root canal treatment training and they were very useful*,* especially initially as teeth were transparent and could see ‘pulp tissue’ inside the tooth and could see how files responded to movement and how to use files to removal all pulpal tissue’.*

**Fig. 4:**
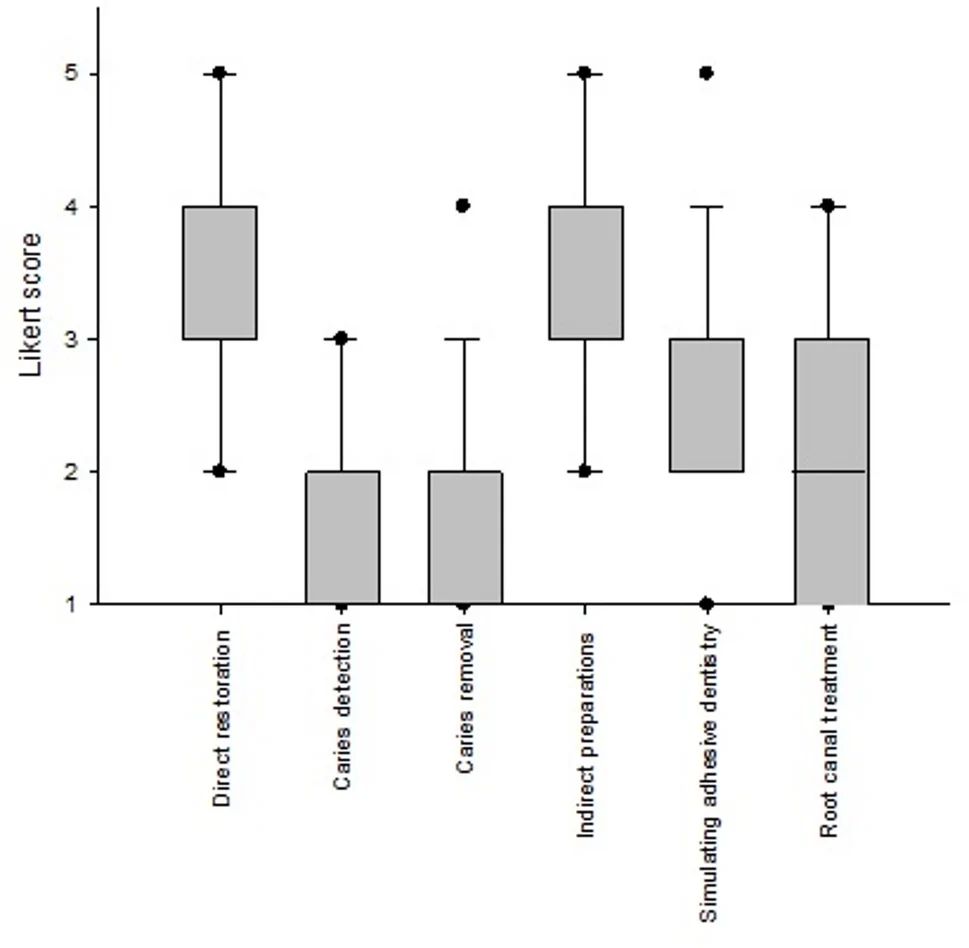
Box and whisker plot depicting the results for Questions 3-8: ‘How effective are the typodont teeth used in your institution at preparing individuals for the following operative procedures in a clinical environment?’. Participants ranked importance: 1 = not very effective at all, 5 = very effective

Root canal simulation effectiveness was viewed negatively overall (M = 2.36 ± 1.12); internal participants (M = 2.23 ± 1.10) (Fig. 4) scored significantly lower than external (M = 2.61 ± 1.13) (*t*(337) = 3.015), *p* = 0.003). The presence of a pulp in a typodont was viewed as important by all (M = 3.92 ± 1.05), as was the presence of root canals for endodontic training (M = 4.15 ± 1.06).

The important link between the successful removal of caries and pulpal health was further highlighted in all experience groups, for example in suggestions for a perfect typodont to: *‘Include pulp*,* allows students to visualise when they have drilled too deep as this is a very common occurrence from the start of clinical years’*, *‘Pulp present so you know if you have exposed’*. A valuable comment from a BDS5 student further explained the importance of this relationship: *‘Typodonts with caries present would provide useful experience for junior students on the extent of tooth tissue to remove*,* as most of this experience is currently gained when treating live patients…not done cautiously*,* this could lead to unnecessary tooth tissue loss and potentially perforations’*. Some comments indicated some issues with advanced endodontic focused typodonts: *‘The 3D printed teeth for RCTs were somewhat similar but the files snapped all the time’* and *‘3D root canal teeth were excellent but melted with heat’.*

### Direct and indirect preparation

Comments valued the ability to repeatedly practice on typodonts: ‘*essential for students to understand the concepts of tooth preparation’*. The advantage of typodonts being readily available was very important to all (M = 4.45 ± 0.84). The overall average for direct cavity preparation simulation effectiveness was moderate (M = 3.56 ± 0.85) with no significant difference between internal (M = 5.58 ± 0.81) and external (M = 5.53 ± 0.92) responses (*t*(337) = -0.52), *p* = 0.602). Likewise, indirect preparation simulation was viewed with moderate effectiveness overall (M = 3.47 ± 0.92); a Mann-Whitney U test indicated no significant difference (*U* = 12,757.50, *p* = 0.639) between internal (Mdn = 3.00) and external responses (Mdn = 4.00).

### Educational experience

The availability of typodonts was rated as an important advantage over extracted human teeth with a mean score of 4.45 (± 0.84): Dunn’s multiple comparisons revealed this advantage was viewed as significantly more important than all other advantages (*p* < 0.001) apart from standardised examinations (*p* = 0.76). All participants appreciated the ability of typodonts to standardise educational experience (M = 4.07 ± 0.99) and examinations (M = 4.25 ± 0.87) (Fig. 3). Mann-Whitney U tests showed no statistically significant difference between student and qualified participants for the standardisation of both educational experience (*p* = 0.097) and examinations (*p* = 0.0694) (U = 24452.00).

Further free-text comments suggest even models with tailored educational qualities have limitations: *‘We used teeth with black caries on a DFT study day. I feel the teeth with black caries unrealistic and pointless*,* you cannot visualise the ADJ being clear you are just removing black colour’* and *‘For RCT we used 3D printed teeth*,* it was helpful for starting out to see what we were doing internally*,* but differs quite significantly from real teeth’*. One comment stated that a perfect typodont was *‘Impossible to make’*.

### Ethical and environmental

Beyond free-text comments suggesting features of a perfect typodont being *‘environmentally friendly’* and *‘recyclable’* there were no specific suggestions of how this might physically be possible. The lack of cross-infection risk was viewed as an important advantage of typodonts (M = 3.90 ± 1.23). Less value was placed, albeit neutral, on the volume of plastic typodonts used from an environmental standpoint (M = 2.82 ± 1.23) and the ethical advantage of typodonts over human teeth (M = 3.34 ± 1.40). However, larger standard deviations were noted in these questions suggesting more diverse opinion. A comment alongside one such ranking explained a potential reasoning: ‘*dentistry already sees a lot of single use plastic wastage*’.

The balance tipped slightly over neutral regard to the importance of a perfect typodont being environmentally friendly and waste limiting (M 3.07 ± 1.20). A Mann-Whitney U test indicated that qualified dentists rated the importance of typodonts being environmentally friendly and waste limiting significantly higher than students did (*U* = 21,051.00, *p* = 0.026). Individual t-test indicated qualified participants (M = 3.11 ± 1.23) also showed more concern over the volume of plastic teeth in use than the student participants (M = 2.61 ± 1.20) (*t*(337) = -3.3.687, *p* < 0.001). All participants shared moderate concern over the potentially harmful effects of 3D-printing resins (M = 3.44 ± 1.17).

## Discussion

Key findings from this study confirm that whilst typodonts are perceived to perform satisfactorily in the repeated practice of direct and indirect preparation tasks, educators and students from multiple institutions agree in their opinion that they do not adequality simulate realistic appearance, caries and endodontic procedures or provide appropriate tactile feedback.

Whilst natural tooth colour was not important for typodont success, the layering of simulation teeth certainly was. Despite a lack of literature on the subject, the educational benefit of using anatomical layers has been confirmed both by those researching commercially available typodonts [3] and those making typodonts in-house [17]. Findings from this questionnaire suggest that advanced typodonts are available but reserved for adjunctive teaching in specialty areas. Question 34 requested information on experience on more ‘advanced’ typodonts; the free-text comments identify their use predominantly in caries and endodontic simulation, rather than direct and indirect preparation exercises. This is confirmed in associated literature: typodonts made in-house by educational institutions seem to fulfill a specific educational need rather than the entire typodont stock [22–25].

The need for better tactile feedback was a high value theme identified by all experience levels in this study, and aligns with previous studies [3, 9, 10, 26, 27], with the majority focusing on questionnaire feedback following usage of small groups of commercially available typodonts or materials. Tactile feedback is a critical sense not only for effective caries removal and tooth preparation but also a minimally invasive approach. It is logical that a typodont should mimic the tactile feedback of the human tooth it aims to simulate, enabling worthwhile practice before treating patients. Typodonts are often criticised for not feeling hard enough [10], which may in turn result in excessive tooth tissue loss through excess pressure – the opposite of minimal intervention. Mimicking this ‘feel’ of human tooth tissue is uniquely difficult given the complexity of the interplay between the operator, the handpiece and drill set, and the mechanical properties of both enamel and dentine, with the latter being particularly challenging. Previous studies confirm the materials used for typodonts do not accurately represent the unique mechanical properties of enamel and dentine [28]. Two groups have analysed average force measurements during automated cutting to attempt to quantify part of this interplay [29–31]. The results led one group on to modifying a commercial 3D-printing resin in order to attempt to improve the mechanical properties of both traditional and advanced typodonts [29, 30], although further improvements in tactile feedback were still required following evaluation.

Some institutions have invested in virtual reality (VR) simulators for operative dentistry to reduce pressure on the use of physical typodont teeth and to potentially overcome some of their limitations. VR simulators have been reported in the literature to be advantageous in their ability to provide assessment metrics and unlimited repetition [32, 33]. However, their use is often still as an adjunct to typodont teeth, with limitations to overcome such as a high cost and their own shortcomings in tactile sensation with respect to recreating the feel of dentine and enamel [34].

Results suggest that typodonts are viewed poorly in their effectiveness in simulating the human tooth with regards to endodontics. As with tactile realism, endodontic simulation has not been perfected even with advanced models, with explanations relating to material properties not being able to replicate the unique properties of enamel and dentine and the complex anatomy of the root canal system [35]. One free text comment highlighted an issue not yet reported in the literature, that no typodont tooth replicates the colour ‘map’ of the pulpal floor, an invaluable feature to root canal identification. Yet, a significant proportion of advanced and 3D-printed models exist to serve Endodontic training. Studies evaluating individual models have also highlighted issues with specific endodontic typodonts such as poor tactile feedback, file breakage and melting with heated obturation techniques with some students still preferring to use extracted teeth despite their disadvantages [36, 37].

Caries simulation was identified as clinically important but not satisfactory, particularly with reference to pulpal exposure. Free-text comments supported this finding with many participants suggesting caries be better represented in typodonts, particularly before commencing clinical work. Some research groups have focused on creating tailored caries typodonts for caries simulation presumably to overcome this gap in the commercial market, requiring considerable time and resource [8, 10, 38]. Other student opinion studies have identified caries simulation as a favoured focus for typodonts [4, 39]. No matter the hours spent in simulation, the transition to clinical practice is not without risk; the risk of pulpal exposure through caries removal was anecdotally important amongst dental educators at UoB and was verified by this data as an issue amongst many institutions. Anxiety and stress amongst students caused by the prospect of pulpal exposure has previously been documented [40] and may partly explain why caries simulation was so heavily requested in the free-text comments by students.

Despite shortcomings in many areas such as root canal simulation and caries, it considered that most typodonts provide moderately effective simulation of both indirect and direct preparations. The predominant use of monoblock typodonts does limit the experience of some users to the potential benefits of advanced models, but reviewing this alongside their limitations of availability and cost explains why there has not been mass adoption of potentially improved models.

The availability of typodonts for repeated use was the most highly rated feature of typodonts, thus the balance between mass availability and price is paramount. It is difficult for an institution to know whether it is wise to invest time, energy and (existing or new) personnel in the development of their own typodonts despite the reported potential advantages, especially if they still do not match up to student expectation. Although exciting, this rapidly developing technology is acknowledged to not be without disadvantages such as high set-up cost and a steep learning curve to using the technology beyond the plug-and-play settings. Printing an object with commercial resins using prescribed settings is relatively straightforward. However, should an institution want to, for example, create typodonts from CBCT scans or formulate resins with specific chemical and mechanical properties, it requires significant expertise amongst its staff.

Despite a variety of locations and resultant funding differences, no participant in this study seemed to have found the perfect typodont. It could be that the topic is too niche for 3D-printing companies to produce a typodont specific resin, and/or that the material properties of dentine and, in particular, enamel are indeed too difficult to replicate irrespective of budget. Advanced typodont models were the minority type in use globally. It is reasonable to assume that the cost of more advanced models is a prohibitive factor limiting their widespread use in many of the institutions. Reliable purchasing data was available only internally, but provides context as to what a typical dental institution spends on simulation education. Typodonts are included in tuition fees for many but not for all, yet surveyed students did not seem to be directly concerned by typodont pricing. Those involved in dental education agreed that cost was important. Cost-benefit analysis of typodont simulation is rarely presented in the literature but some studies confirm that production of in-house 3D-printed typodonts is a viable option for some institutions; cost is sometimes mentioned in the literature as ‘cost per tooth’, more rarely with additional brief mention of equipment expense and cost-per-tooth, but never to the authors best knowledge with a full cost-benefit analysis [22, 23, 25, 27, 41]. Initial costs of setting up 3D-printing facilities may over time be more cost-effective than a yearly commercial typodont budget; however, this will be specific to the finances of each institution and requires in-depth analysis.

Alongside cost, there was moderate concern over the environmental impact of the 3D-printing resins used for newer advanced models with qualified dentists showing greater concern: experience (and age) may lend itself to increased concern over the environment relative to the known volumes of typodonts used. It is reasonable that students were more concerned by realism and tactile feedback than the financial and environmental impact, whereas educators might consider broader implications having obtained their primary qualification. It is also acknowledged that opinion over concern for the environment can be polarising. There was overall agreement that typodont teeth should aim to be environmentally friendly and waste-limiting with suggestions for improvement being superficial without specific ideas of how this might be possible.

At the time of writing, there is no commercially available 3D-printing resin for typodonts, with mostly generic commercially available resins used in studies that have printed their own typodonts [17, 42, 43] .The authors only know of one research group in the literature preparing filled 3D printable resins specifically for typodonts, but this still uses a commercially available photopolymer base with added filler particles [30, 35]. Commercially available liquid photopolymer resins used in SLA and DLP 3D-prinitng can be bought online and used by the public. Review of the associated Safety Data Sheets for the liquid 3D-printing resins commonly in use for in-house typodont printing indicate risk to aquatic life, eyes, skin and the reproductive system. These risks decrease significantly once the printed typodont undergoes a post-cure protocol, yet uncured resin is accessible to the user at every stage of production.

Review of studies documenting in-house typodont production simply do not mention the environmental or health and safety impact of the liquid 3D printing resins they use. German dental students are required to purchase their own typodonts; the research group at the University of Würzburg, suggest that students could reduce cost burden by printing their own typodonts thus handling resins in their liquid state [27]. Only one study was found to mention designed reusability of in-house manufactured typodonts as an environmental advantage [25]. Additive manufacture provides significant potential waste-limiting and reduced energy consumption advantages [44] yet it is the authors’ opinion that the community should seek use of environmentally responsible resins. Although a small contributor to the monumental amount of single-use plastic in healthcare, it does not mean it should be overlooked and may prompt others to pursue innovation.

### Limitations

The themes identified are intended to give a broad overview of the use of typodont simulation across multiple institutions, building upon the existing literature of smaller studies investigating specific limitations and typodonts. It is important to consider that the variety of exposure to different typodonts and curricular variances across institutions will have influenced participant perceptions and so conclusions are cautious and not generalisable.

Despite efforts to share the questionnaire with as many professional colleagues as possible, it is acknowledged that some areas were not reached as effectively. Translation of the questionnaire into multiple languages would have increased response rate and been more inclusive. The sample size could have been bigger; however, results of this study can be viewed with statistical power owing to response rates in excess of those required by power calculations. The majority of responses are from UoB and thus provides a source of bias. The study should be viewed as exploratory rather than conclusive worldwide evidence, given the difficulty recruiting international participants within the capabilities of this study. The recruitment process may have introduced selection bias favouring those who may be more familiar with 3D-printed typodonts. The analysis of multiple Likert-scale responses may have increased the risk of Type I error.

Questions relating to environmental factors such as volume of typodonts used and on environmental concerns were perhaps leading questions by providing contextual information. However, participants may otherwise not have been informed enough to answer these questions: it was assumed that prior knowledge on quantities of typodont orders and chemical composition of liquid 3D-printing resins was low, and information provided was factual.

### Educational impact

Previous studies have evaluated typodont simulation but often focus on one brand or type, and often with small cohorts of internal participants. Whilst these studies are a useful part of niche research and outliers in advanced education, the comparatively considerable number of responses from this questionnaire validates important viewpoints on the predominant typodont simulation.

Participants in this study agree that whilst they have some perceived benefit to educational experience. This study confirms the high value that our students place on standardisation in education and assessment, which typodonts can achieve, as well as targeting specific learning needs in areas of self-identified low confidence. The perceived importance of case-specific typodonts was particularly valued by more experienced students: a reflection of their dental degree, migrating from single-item to entire case management.

Evaluation of such current simulation methods is critical to allow development of clinical confidence, for both improved educational delivery and patient safety. Improved simulation has previously been concluded to improve standards of care for patients [5]. Tailored exercise typodont simulation has also been linked to a reduction in student stress and anxiety and an improvement in confidence [40], which is an important consideration for all institutions. Some studies have identified an improvement in student engagement and staff opinion with use of specialised typodonts [27].

### Future research and recommendations

Future research in this area should aim to develop methods of production of typodonts, utilising the potential benefits of additive manufacturing such as customisation, local made-to-order manufacture and cost-effectiveness. Institutions should also target areas where current simulation offerings are sub-standard, to include tactile feedback, caries and endodontic simulation, with cost and the environment in mind. Institutions should of course follow health and safety regulations surrounding 3D-printing: despite 3D printers being readily available to the public, there are key environmental and safety considerations. For those interested in designing and creating complex models, there is a steep learning curve for those unfamiliar with the process.

So-called ‘environmentally friendly’ resins exist, either produced via naturally occurring substances and organic feedstocks or that they are post-processed using water and not solvents [44, 45]. However, greenwashing (the act of deception over environmental friendliness) should be closely scrutinized. There are no case studies or original research articles using 3D-printed typodonts that have assessed these types of resins in this field. This would be a prudent development alongside further quantitative analysis of the tactile feel of typodonts to complement the largely qualitative bank of research in the area.

Whilst 3D-printing is rapidly evolving, it is essential that innovation remains focused on enhancing the quality and educational value of models. Typodonts must be tested by those that teach dentistry, evaluated by dental students and experienced clinicians, to ensure they are fit-for-purpose. It would be prudent to repeat effectiveness evaluations following development of design and functionality to elucidate whether quality is improving over time.

## Conclusion

In the opinion of students and qualified dentists across multiple institutions, no typodont appears to excel in their appearance, tactile feedback and simulation of caries, root canal treatment, direct and indirect preparation simulation. There is perceived value of educational impact of typodont simulation despite their limitations, with improvements possible to enhance education further. There was varied opinion on the environmental impact of typodont teeth but a perceived benefit to its consideration in future developments. This comprehensive study of opinion revealed that anatomical layering, tactile realism and accurate caries reproduction were highly valued features of typodont simulation to both students and qualified dentists but are poorly represented in the predominant commercially available options. There are more advanced typodonts produced, used by the minority, but they still have limitations given the complexity of replicating human tooth properties. Future work should combine both materials science and educational research to expand the possibilities for local production of custom typodonts. It is likely that 3D-printing innovation will continue to develop and fill these gaps in simulation, thereby enhancing dental education for those with expertise. Environmental impact, financial viability and mass availability should not be overlooked.

## Data Availability

The datasets generated and/or analysed during the current study are not publicly available as it forms part of an ongoing doctoral thesis. In the meantime, datasets are available from the corresponding author on reasonable request.

## Abbreviations

3D: Three dimensional
BDS: Bachelor of Dental Surgery
DLP: Digital Light Processing
M: Mean
Mdn: Median
QD: Qualified Dentist
QDE: Qualified Dentist and Educator
SLA: Stereolithography
U: Mann-Whitney U value
UoB: University of Birmingham
